# Editorial: Recent advances and new challenges in minimally invasive surgery and chemotherapy for colorectal cancer-volume II

**DOI:** 10.3389/fsurg.2025.1665001

**Published:** 2025-08-07

**Authors:** Hiroki Hashida

**Affiliations:** Department of Gastrointestinal Surgery, Hanwa Memorial Hospital, Osaka, Japan

**Keywords:** colorectal cancer, chemotherapy, minimally invasive surgery, laparoscopic surgery, robotic surgery

**Editorial on the Research Topic**
Recent advances and new challenges in minimally invasive surgery and chemotherapy for colorectal cancer-volume II

Following the success of Volume 1 of this Research Topic, which highlighted foundational progress and innovative approaches in the surgical and chemotherapeutic management of colorectal cancer (CRC), we are pleased to present Volume 2. Building upon the insights and momentum established in the first edition, this second volume continues to explore the frontiers of minimally invasive techniques, multimodal therapies, and emerging research paradigms in CRC treatment.

Colorectal cancer remains a major cause of global cancer mortality. With advances in surgical technology and medical oncology, treatment is becoming increasingly tailored, multidisciplinary, and data-driven. The articles in this collection reflect this evolution—offering both clinical evidence and conceptual frameworks to address persistent challenges in CRC care.

A prominent theme in this volume is the refinement of neoadjuvant therapy strategies for locally advanced rectal cancer. Zhou et al. conducted a comprehensive systematic review and meta-analysis comparing neoadjuvant chemotherapy (NCT) alone vs. long-course chemoradiotherapy (Lc-NCRT). Their analysis of over 5,000 cases suggests that NCT may provide similar long-term outcomes with fewer postoperative complications, such as anastomotic leakage and permanent stoma formation, raising important considerations for organ preservation and patient quality of life.

Complementing this, Foroughi et al. reported results from a phase 3 randomized clinical trial comparing total neoadjuvant therapy (TNT) to the standard sequence of chemoradiation followed by surgery and adjuvant chemotherapy. TNT was associated with higher pathological complete response rates without increasing treatment toxicity, underscoring its value in comprehensive local and systemic disease control.

The role of robotics in minimally invasive colorectal surgery continues to expand, as discussed in two key contributions. In a single-center comparative study, Wang et al. found that robotic-assisted surgery, while associated with longer operative times and higher costs, achieved similar clinical outcomes to laparoscopy in terms of complications, hospital stay, and oncologic integrity. Chen et al. offered a broader perspective with a narrative review detailing the clinical advantages of robotic surgery and its integration into multidisciplinary treatment pathways, while highlighting current limitations such as lack of tactile feedback and economic constraints.

Accurate risk prediction remains a cornerstone of personalized oncology. Huang et al. developed a clinical nomogram and web-based calculator to predict local recurrence and overall survival following radical surgery for stage II–III CRC, facilitating more precise postoperative planning and follow-up.

The dynamic interplay between local treatment and systemic immune modulation in colorectal liver metastases was explored through a bibliometric analysis by Shao et al., revealing growing interest in combining local interventions such as ablation or transarterial therapies with immunotherapy, particularly in the context of oligometastatic disease.

Altogether, this second volume underscores the continued innovation, diversification, and integration across surgical and medical disciplines in the management of CRC. While the data presented offer significant promise, they also reinforce the need for ongoing clinical trials, cost-benefit analyses, and the standardization of protocols—particularly in robotic surgery and neoadjuvant regimens.

We would like to thank all authors, reviewers, and editors for their scientific contributions and collaboration. In particular, we extend our sincere gratitude to Professor Nobu Oshima for his valued support and dedication as co-editor of this Research Topic. His contributions were instrumental in ensuring the quality and cohesion of this collection.

We hope this volume serves as both a reference and an inspiration for future studies aiming to optimize the treatment and outcomes of patients with colorectal cancer.

